# NAFLD, MAFLD, and beyond: one or several acronyms for better comprehension and patient care

**DOI:** 10.1007/s11739-023-03203-0

**Published:** 2023-02-17

**Authors:** Piero Portincasa

**Affiliations:** Clinica Medica “A. Murri”, Department of Preventive and Regenerative Medicine and Ionian Area (DiMePrev-J), University of Bari Aldo Moro, Piazza Giulio Cesare 11, 70124 Bari, Italy

**Keywords:** Cardiovascular disease, Diabetes, Endocrine, Fatty liver, Hepatic fibrosis, Hepatocellular carcinoma (HCC), Insulin resistance, Metabolic syndrome, Obesity, Steatosis

## Abstract

The term non-alcoholic fatty liver disease (NAFLD) has rapidly become the most common type of chronic liver disease. NAFLD points to excessive hepatic fat storage and no evidence of secondary hepatic fat accumulation in patients with “no or little alcohol consumption”. Both the etiology and pathogenesis of NAFLD are largely unknown, and a definitive therapy is lacking. Since NAFLD is very often and closely associated with metabolic dysfunctions, a consensus process is ongoing to shift the acronym NAFLD to MAFLD, i.e., metabolic-associated fatty liver disease. The change in terminology is likely to improve the classification of affected individuals, the disease awareness, the comprehension of the terminology and pathophysiological aspects involved, and the choice of more personalized therapeutic approaches while avoiding the intrinsic stigmatization due to the term “non-alcoholic”. Even more recently, other sub-classifications have been proposed to concentrate the heterogeneous causes of fatty liver disease under one umbrella. While awaiting additional validation studies in this field, we discuss the main reasons underlying this important shift of paradigm.

**Supplementary Information:**

The online version contains supplementary material available at 10.1007/s11739-023-03203-0.

## Introduction

The acronyms NASH (non-alcoholic steatohepatitis) and NAFLD (non-alcoholic fatty liver disease) were originally coined by Ludwig et al. in 1980 [1] and Shaffner and Thaler in 1986 [2], respectively. NAFLD is a clinico-histopathologic entity defining excessive hepatic fat storage without evidence of secondary hepatic fat accumulation [3–7] in patients with “no or little alcohol consumption”. Hepatic fat content must be greater than 5% at liver histology [8] or 5.6% at magnetic resonance [9]. 

Since 2020, a debate is taking place worldwide to redefine NAFLD as metabolic (dysfunction)-associated fatty liver disease (MAFLD), according to the most prevalent causes of liver steatosis [10–12]. Meantime, further terminologies are being proposed instead of NAFLD. While awaiting additional validation studies, the main reasons underlying this important shift of paradigm are discussed in the following paragraphs.

## NAFLD and the burden of disease: prevalence, natural history, etiology and pathogenesis

Globally, NAFLD has reached epidemic levels, with pooled prevalence of 14% (Africa), 24% (North America) [13], 24–27% (range 18–40%, Europe) [14], 27% (Asia), 31% (South America), and 32% (Middle East) [13]. NAFLD is more frequent in men than in women (33 vs. 20%), and also affects 10–20% of the pediatric population [15]. Evidence indicates the prevalence of NAFLD is on the rise worldwide together with that of obesity and associated complications [11, 16, 17]. 

In this scenario, we can expect a time-dependent increase of the incidence of liver fibrosis, decompensated liver cirrhosis, hepatocellular carcinoma, and liver-related mortality due to the progressive deterioration of initial NAFLD in the affected population [17].

**Fig. 1:**
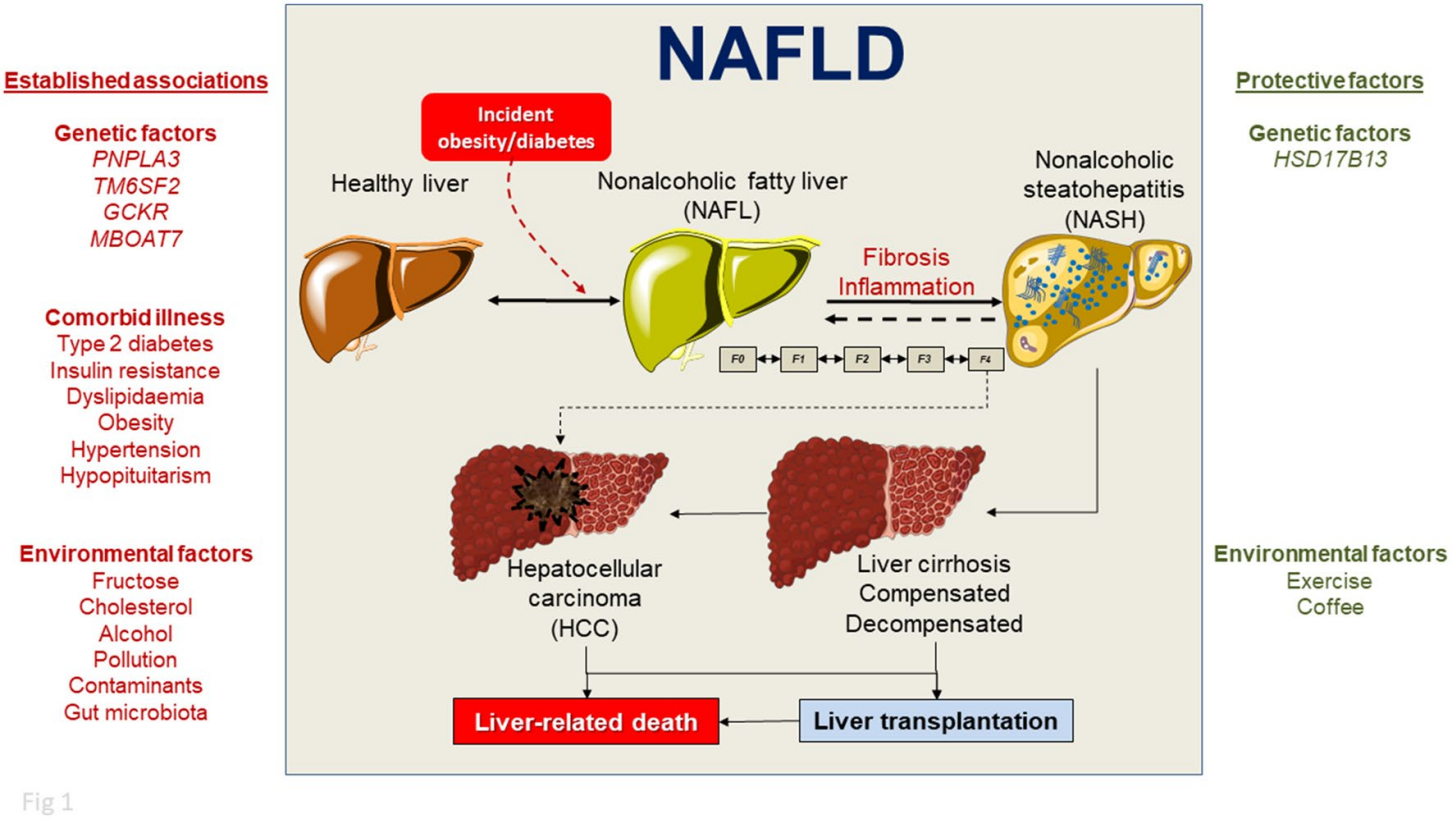
Causal factors, protective factors and the continuum spectrum of natural history of non-alcoholic fatty liver disease (NAFLD). Factors on the left have an established association with NAFLD and NASH progression. They are broadly classified into genetic factors, comorbid illness, and environmental factors. On the right, factors have a protective role. *F0–F4*, fibrosis scores (potentially reversible)

The natural history of NAFLD can vary, with most patients developing a benign or slowly progressive form, which is usually asymptomatic in the early stages. The spectrum ranges from simple steatosis (non-alcoholic fatty liver, NAFL) to the progressive non-alcoholic steatohepatitis (NASH) in about 20% of cases [18]. NASH has the potential to progress to compensated and decompensated liver cirrhosis and, with more than a tenfold increase in risk, to hepatocellular carcinoma (HCC) (Fig. 1) [9, 19]. Of note, NAFLD is also a risk factor for several extrahepatic, especially metabolic manifestations [20] and is linked with increased cardiovascular risk [21].

Excessive liver fat storage has a series of well-known causes (Supplementary Table 1) and originates from a dynamic balance between causal and protective factors (Fig. 1) [22, 23]. 

In this scenario, individual lifestyle changes and environmental factors can promote epigenetic mechanisms such as histone methylation, abnormal DNA methylation, miRNA profiles, able to affect gene expression and to influence the progression of disease. Examples are the consequences of metabolic disturbances in pregnancy on NAFLD offspring [24], and the pro-inflammatory liver response and weight gain in germ-free mice colonized with stool microbes from 2-week-old infants born to obese mothers [25]. These mechanisms interact with inherited risk factors and modulate individual susceptibility to NAFLD [26].

Lifestyle and dietary habits [27, 28] play a key role, and NAFLD is commonly associated with metabolic abnormalities [9] such as obesity, type 2 diabetes, dyslipidemia, hypertension, hypopituitarism, and sedentary life [20]. On the other hand, alcohol, air pollution [29, 30], food contaminants [31], and gut dysbiosis, e.g., higher proportion of *Proteobacteria* and *E. coli*, with a lower proportion of *Firmicutes*, especially *F. prausnitzii* [32] likely contribute to the onset and progression of NAFLD [20, 33, 34].

In the context of metabolic abnormalities, several pathogenic pathways are involved in lipotoxicity and can contribute to the onset and progression of NAFLD, according to the nomenclature in use. The “lean” healthy visceral adipose tissue expresses anti-inflammatory cytokines (i.e., adiponectin, interleukin IL-4, IL-10, IL-13, transforming growth factor (TGF)-β, and nitric oxide (NO)) which control M2 macrophagic response and inhibit the neutrophil-mediated inflammation. By contrast, during expansion of hypertrophic (and apoptotic) visceral adipose tissue, secretion of pro-inflammatory molecules such as leptin, resistin, IL-6 and tumor necrosis factor (TNF)-α occurs. This step activates a M1 macrophage response [35], and results in insulin resistance, a chronic “metabolic” inflammatory status, and increased lipolysis of triglycerides with abundant flux of blood long-chain free fatty acids (FFA) to the liver. In addition, FFA in blood increases because of fat-enriched dietary habits and from dietary sugars driving the de novo lipogenesis (DNL) in the liver. Altogether, these factors contribute to the expansion of the hepatocyte FFA pool which can stress the mitochondrial β-oxidation capacity, lead to defective secretion/export of very-low density lipoproteins (VLDL) to blood. Accumulation of lipotoxic species such as lysophosphatidylcholine, diacylglycerol, and ceramides mediates the endoplasmic reticulum (ER) stress, the cellular oxidative stress, and the activation of the inflammasome. This is a component of the innate immunity response consisting of a multiprotein cytoplasmic complex activated by several damage-associated molecular patterns (DAMPs). Additional abnormalities consist of dysregulation of adipocytokines, depleted mitochondrial ATP, production of toxic uric acid, periodic hypoxia (i.e., during sleep apnea in extremely obese patients), and toxic products from gut microbiome which include tumor necrosis factor (TNF)-α, endogenous ethanol, and endotoxins like lipopolysaccharides (LPS). Studies in pure fatty liver models without fibrosis suggest that lipid accumulation developing with obesity can induce a distortion of liver architecture manifesting with reduced sinusoidal space and increased intrahepatic vascular resistance. Such changes can pave the way to portal hypertension observed in obesity, and progression to hemodynamically decompensated liver cirrhosis [36–39].

Conditions can promote the NASH phenotype which manifests with hepatocellular injury, inflammation, stellate cell activation and progressive accumulation of excess extracellular matrix. Additional targets of the ongoing cellular damage include intracellular organelles, the nucleus, receptors and signaling pathways [40–44].

### NAFLD and metabolic dysfunctions

As mentioned earlier, the diagnosis of NAFLD is based on hepatic steatosis at imaging techniques or histology, and “exclusion” of competing causes of liver disease including “significant” alcohol intake. Such strict definition has some limitations. Despite the NAFLD prognosis depends on the presence of fibrotic NASH, the ultimate utility of liver biopsy becomes questionable [45]. In fact, liver biopsy is invasive, painful, risky, prone to misclassification due to sampling errors, not easily performed in large groups of patients who still lack targeted therapies for NAFLD. The issue of alcohol intake in NAFLD deserves additional observations. A standard drink contains about 14 g of pure alcohol (Rethinking Drinking Homepage—NIAAA (nih.gov)) and the current definition for NAFLD must exclude a weekly intake of ≥ 21 and ≥ 14 drinks in males and females, respectively. Above this cutoff value, the risk of alcoholic fatty liver disease (ALD) increases [46, 47], but it is difficult to exactly calculate the intake of alcoholic units or the duration of alcohol abstinence. Phosphatidylethanol can become a potential biomarker of alcohol consumption [48]. Yet, the effect of modest alcohol intake at lower cutoff values is still controversial in NAFLD individuals [49, 50]. 

In addition, the diagnosis of NAFLD requires the exclusion of several other causes of liver steatosis such as viral hepatitis [51–54], hepatotoxic drugs [55], Wilson’s disease [56], total parenteral nutrition, prolonged fasting [57], and several other less common conditions (Supplementary Table 1). Both NAFLD and ALD rank as the most frequent conditions [58]. On one hand, the diagnosis of NAFLD relies on exclusion criteria and does not require the presence of metabolic dysfunction. But NAFLD is no longer an isolated condition, since NAFLD is associated with morbid obesity in about 90% of the cases [59, 60], with obesity and dyslipidemia in over 80% of the cases [61, 62], with hypertension in 70% of cases and with type 2 diabetes (T2DM) in about 50% of the cases [63–65]. Such close associations strongly suggests that NAFLD is a systemic disease [10, 11, 15, 66] increasing with poor lifestyles, and in parallel with the epidemiological raise of overweight, obesity, insulin resistance, and metabolic syndrome [4, 12, 67–70]. Younossi et al. [71] studied the prevalence of NAFLD and NASH in a metanalysis including 80 studies from 20 countries worldwide and 49,419 patients with T2DM. The prevalence of NAFLD was more than twofold higher than in the general population, i.e., 56%. In the same group, the prevalence of NASH was 37%. In patients with NAFLD and T2DM undergoing liver biopsy, 17% had advanced (F3–F4) liver fibrosis [71]. The close association of NAFLD with systemic metabolic conditions also explains why cardiovascular complications are frequent in NAFLD patients [65, 72], including atrial fibrillation [73, 74], diabetes, chronic kidney disease and extrahepatic neoplasms [75–77]. It is worth noting that 10–30% of non-obese individuals can have NAFLD [4, 78].

Another close link between NAFLD and metabolic dysfunction is that the metabolic syndrome is often associated with NAFLD and with increased cardiovascular disease. NAFLD per se*,* however, is independently associated with cardiovascular disease [65, 72, 79, 80].

The ultimate knowledge of complex mechanisms governing the onset and progression of NAFLD is poorly known. The term “steatosis” is intrinsically characterized by the interplay of multifactorial factors [34, 40, 81, 82]. The coexistence of such factors makes the diagnosis of NAFLD and the design of clinical trials often difficult, since several confounding factors can be present [9]. 

### MAFLD: debate about nomenclature

The acronym NAFLD was originally established as a diagnosis of exclusion based on the use of the stigmatizing term “alcoholic” and on a poor pathophysiological knowledge. In the past years, this term has generated confusion or uncertainty with regard to knowledge and allocation in clinical trials [83, 84]. Studies show that up to 96% of subjects with NAFLD can be unaware they have liver disease [85]. 

**Fig. 2:**
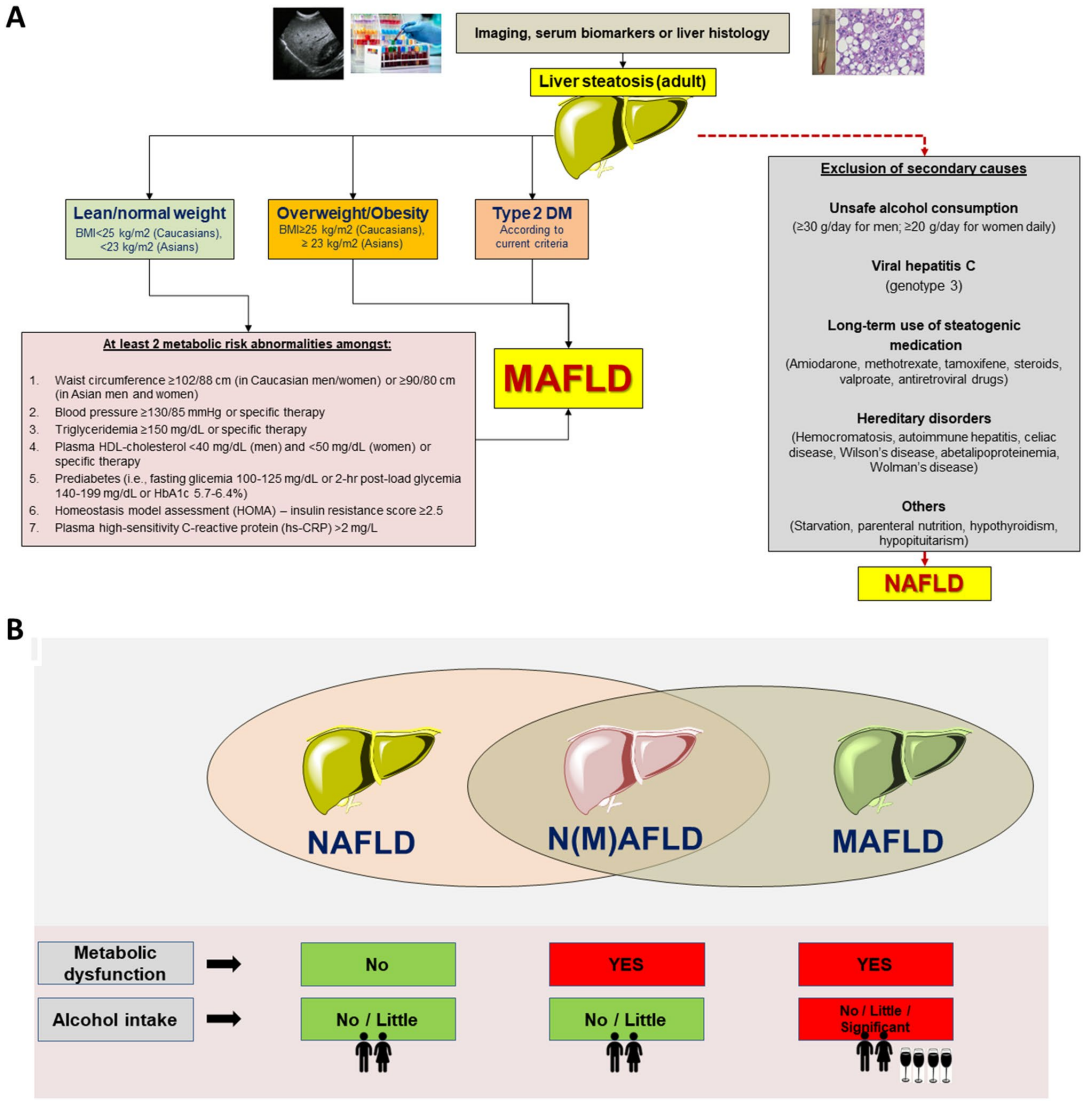
**A** The flowchart depicts the essential steps involved in the positive diagnosis of MAFLD vs. the diagnosis of NAFLD which requires the exclusion of secondary causes. Adapted from [10, 134]. **B** Exclusive and overlapping features in the spectrum of definitions ranging from non‐alcoholic fatty liver disease (NAFLD) to metabolic-dysfunction‐associated fatty liver disease (MAFLD). Significant alcohol intake is ≥ 30 g/day and ≥ 20 g/day in men and women, respectively

Following the current directions, there is a need for adopting a better term for liver steatosis to emphasize what the disease “is”, rather than what “it is not” when considering the burden of contributing metabolic abnormalities, pathophysiological mechanisms, diagnostic, and therapeutic strategies [84]. Since 2020 experts from 134 countries have proposed the transition of terminology from NAFLD to metabolic dysfunction-associated fatty liver disease (MAFLD) [10] which points to the close association between fatty liver, metabolic disorders and target organ dysfunction (i.e., diabetes, chronic kidney disease, atherosclerosis, lung dysfunction, colon cancer, and both intrahepatic and extrahepatic events) [86], rather than on exclusion criteria [12]. The new terminology is not yet endorsed by international societies, including European Association for the Study of Liver Disease (EASL) and the American Association for the Study of Liver Disease AASLD) [87]. MAFLD is based on the presence of fatty liver at imaging, or by the combination of serum biomarkers or liver biopsy (Table 1) in individuals with overweight/obesity (different in Caucasians or Asians), T2DM (i.e., HbA1c ≥ 6.5% or specific drug treatment). In lean/normal weight individuals, the diagnosis is based on the presence of metabolic dysregulation with at least two out of seven abnormalities which include increased waist circumference, blood pressure, hypertriglyceridemia, low plasma high-density lipoprotein cholesterol, impaired fasting plasma glucose, insulin resistance with impaired Homeostatic Model Assessment of Insulin Resistance, and subclinical inflammation by plasma high‐sensitivity C‐reactive protein level [10, 83, 88–92] (Fig. 2A).

**Table 1:** Diagnosis of liver steatosis/fibrosis/inflammation/progression

Outcome	Methods		Notes
Steatosis	Serum biomarkers	AST, ALT, GGT	Not specific, can fluctuate or remain normal during progression of disease
	Algorithms	Hepatic Steatosis index Fatty Liver Index	Combined serum tests and metabolic information [110, 129]
	Imaging	Abdominal ultrasound	Easily available, noninvasive Low sensitivity for steatosis degree < 30%
		ATI	To be validated [130]
		MRI-PDFF	Expensive, limited availability
	CAP	Software (component of transient elastography)	Quantitative measurement of liver steatosis [131]
Fibrosis	Serum biomarkers	FIB-4, NFS, ELF	Combined anthropometric data and serum tests. Can exclude advanced fibrosis [132]
	Imaging	TE	Validated, accurate, associated with measurement of liver stiffness and steatosis (CAP). Decreased sensitivity in obese patients [133]
		2D-SWE	Liver stiffness
		MRI-MRE	Expensive, limited availability
Steatohepatitis	Imaging	SWDS	To be validated [130]
Progression of disease	Liver biopsy	Steatosis, fibrosis, inflammation	Invasive, limitations (see paragraph)

*ALT* alanine aminotransferase, *AST* aspartate aminotransferase, *ATI* attenuation imaging, *CAP* controlled attenuation parameter, *2D-SWE* two-dimensional shear wave elastography, *ELF* enhanced liver fibrosis test, *GGT* γ-glutamyl transferase, *MRE* magnetic resonance elastography, *MRI* magnetic resonance imaging, *NFS* NAFLD fibrosis score, *PDFF* proton density fat fraction, *SWDS* shear wave dispersion slope (SWDS), *TE* transient elastography

According to this perspective, the adoption of MAFLD definition provides a better classification of patients at higher risk of cardiovascular and kidney diseases [21, 93–95], and hepatic fibrosis [96–98], independently of other causes of liver damage. For example, MAFLD terminology becomes independent of alcohol intake, a situation which appears to worsen hepatic fibrosis and increase mortality in liver steatosis [99–103]. On the other hand, one could argue that liver disease in general is often associated with alcohol abuse in the perception of the general public, and that adding “non-alcoholic” would be beneficial since it is now explicitly stated that the liver disease is not related to alcohol.

The presence of viral hepatitis is not an “a priori” exclusion criterium for MAFLD. In addition, MAFLD represents a further risk factor for HCC in hepatitis C virus (HCV) and hepatitis B virus (HBV) patients [104, 105]. Vice versa, the 10-year risk of cardiovascular disease is higher in patients with MAFLD and concomitant viral infection by hepatitis B or C virus, than in patients with only MAFLD [106]. With MAFLD, moreover, the role of metabolic abnormalities on liver damage will be elaborated together with other causes of liver steatosis, such as drugs, pregnancy and gut surgery [107–109]. This possibility will be labeled as “MAFLD plus additional cause ….” [11, 96]. 

The diagnosis of MAFLD can include the presence of positive biomarkers independently from imaging and histology. This is not the case in NAFLD (see above). Thus, the possibility exists that a patient referred with a high-score for a specific algorithm such as the fatty liver index [110]) will be classified as MAFLD in the presence of metabolic dysfunction.

By contrast, the criteria designed for MAFLD do not extend to individuals with liver steatosis without metabolic dysfunction.

In a recent metanalysis and systematic review involving a pool of over 3 million individuals, the prevalence of MAFLD was 39%, 30%, and 5% in obese, non-obese, and normal weight individuals, respectively. Although not all cases of NAFLD are MAFLD [93, 111], by adopting the MAFLD terminology, the clinicians can better understand the pathophysiological mechanisms of disease. In terms of risk assessment, although evidence is limited, MAFLD (but not pure NAFLD) can be directly related with all-cause mortality [112, 113]. NAFLD has been linked with the development of cardiovascular diseases [114], although this association seems mainly linked with the metabolic components. In fact, NAFLD subjects not classifiable as MAFLD are at lower risk [21, 115]. Therapeutic approaches will improve since MAFLD points to the interaction of several pathophysiological factors and the multidisciplinary collaboration between internists, cardiologists, endocrinologists, nutritionists, hepatologists, and family medicine [10, 11, 83, 91, 94, 115–122]. In addition, the target populations will gain a better comprehension of terminology [88, 123] without feeling stigmatized because of the word “alcoholic” [12, 119, 121, 124]. 

The change in terminology, along with either exclusion or inclusion criteria for NAFLD and MAFLD, respectively, creates three groups partially overlapping (Fig. 2B):

- “Pure” NAFLD (non-MAFLD) where metabolic dysfunction is absent, and significant alcohol intake is excluded.
- “Overlapping” N(M)AFLD where metabolic dysfunction is present and significant alcohol intake is excluded.
- “Pure” MAFLD (non-NAFLD) where metabolic dysfunction is present and alcohol consumption can be significant.

Furthermore, in terms of risk assessment related to metabolic phenotypes (all-cause mortality risk, cardiovascular risk, histological progression of liver disease), subjects with overlapping or pure MAFLD represent a heterogeneous group. We speculate that further studies should assess the suitability of a more detailed risk stratification of MAFLD based on specific metabolic phenotypes (Table 2). Recent evidence in a large Korean cohort reported a higher cardiovascular disease risk in lean MAFLD or MAFLD associated with diabetes mellitus, than in overweigh MAFLD subjects, irrespective of metabolic abnormalities or comorbidities. In this cohort, the cardiovascular risk was linked with advanced liver fibrosis irrespective of MAFLD subtype [125].

**Table 2:** Possible sub-classification of subjects with MAFLD according to metabolic phenotype

1. Normal weight, metabolic unhealthy, without type 2 diabetes 2. Normal weight, metabolic unhealthy, with type 2 diabetes 3. Overweight, metabolic unhealthy, without type 2 diabetes 4. Overweight, metabolic unhealthy, with type 2 diabetes 5. Obese, without type 2 diabetes 6. Obese, with type 2 diabetes	

It is evident that further studies are required to better focus on risk-specific sub-profiles (adjusted for confounders) and natural history of MAFLD/NAFLD association between MAFLD and hypertension and diabetes in the last two groups [126]. Despite few studies have dealt with the comparison between NAFLD and MAFLD, we still need caution due to the nature of the studies (retrospective), selection of groups, statistical issues and conclusions of the studies [96, 127].

### Fatty liver disease: more acronyms on the way

**Fig. 3:**
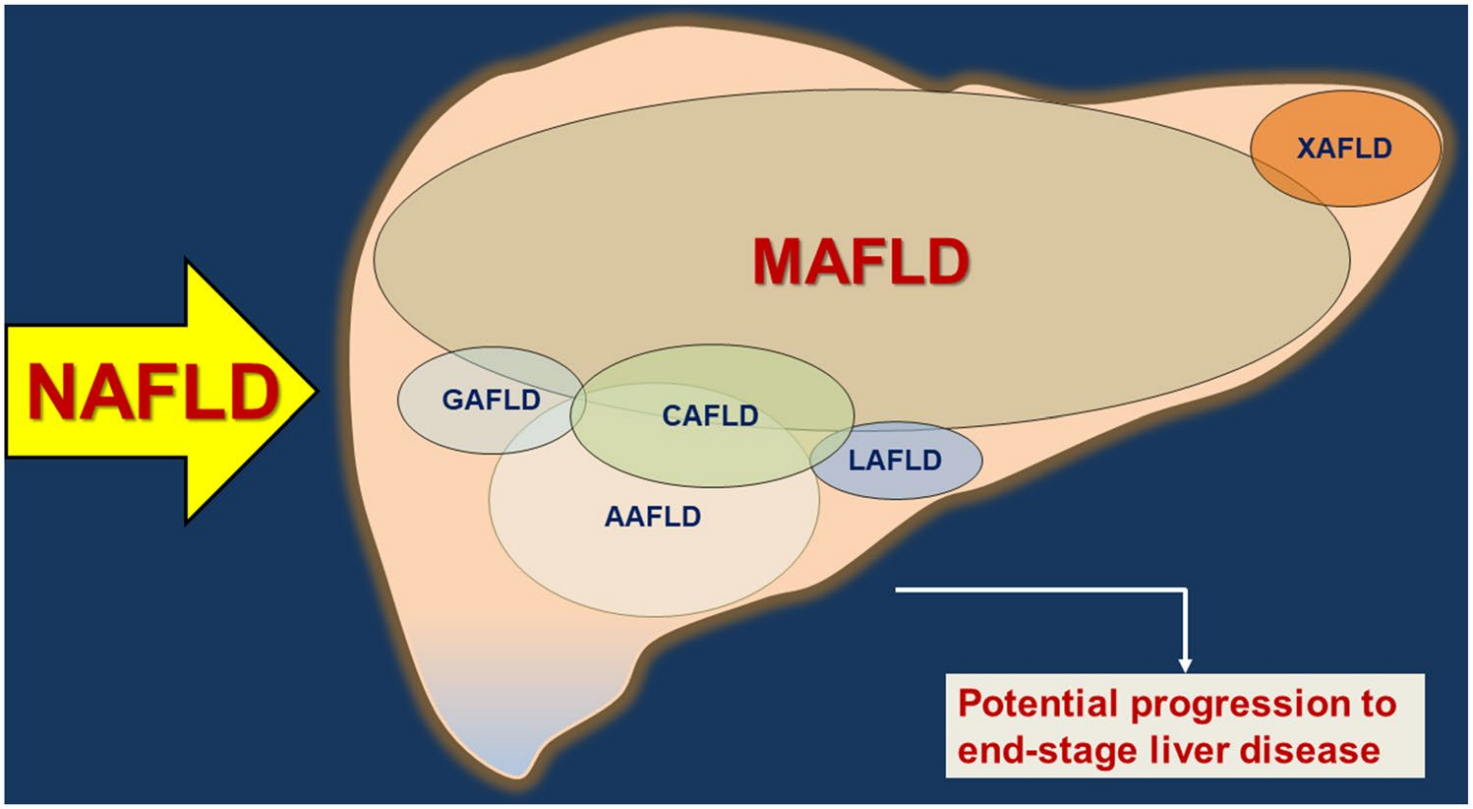
Venn diagrams summarizing the current debate about the nomenclature of NAFLD in relation to other causes of fatty liver disease (FLD). The paradigm shifts from a diagnosis of exclusion («non-alcoholic») to active pathophysiologically established diagnoses involving alcohol abuse, metabolic, genetic, lipodystrophic, combined and yet-to-be-defined causes. *AAFLD* alcoholic-associated fatty liver disease, *CAFLD* combined causes of FLD, *GAFLD* genetics-associated fatty liver disease, *LAFLD* lipodystrophy-associated fatty liver disease, *MAFLD* metabolic-associated fatty liver disease, *NAFLD* non-alcoholic fatty liver disease, *XAFLD* yet-to-be-defined subgroups

Notably, a further classification of liver steatosis is being proposed under the general umbrella of fatty liver disease (FLD). Sub-classifications include almost all possible combinations of genetic, lipodystrophy, metabolic, alcoholic, combined, and yet-to-be-defined causes. This schematic classification will stimulate a further discussion with the aim to improve both comprehension and diagnostic/therapeutic approaches for FLD populations (Table 3) (Fig. 3) [84]. The discussion has gone further with a novel taxonomic classification of NAFLD based on hepatic, pathogenic and systemic features of disease in the individual patient [86, 128]. The liver-determinant-extrahepatic (LDE) system applies to NAFLD and MAFLD and combines information on liver status independently of histology (L), determinants which include sex and reproductive status, genetic, and endocrine assessment (D), and extrahepatic manifestations at a metabolic, cardiovascular, and tumor level (E).

**Table 3:** Recently proposed sub-classifications of fatty liver disease (FLD) [84]

Acronym	Condition	Notes
MAFLD	Metabolic-associated fatty liver disease	Fatty liver because of the overarching metabolic syndrome. Can occur in lean individuals, in overweight/obese individuals, and in T2DM individuals [10–12, 134]
*O-MAFLD*	Obesity-linked MAFLD	Subclassification of MAFLD. Defines the altered gluco-lipid metabolism and pro-inflammatory metabolic changes which link obesity to NAFLD [23, 40, 84, 135, 136]
*SMAFLD*	Sarcopenia-linked MAFLD	Subclassification of MAFLD. Fatty liver occurring during age-related, chronic progressive muscle mass loss, with or without obesity [137]. Sarcopenia can induce insulin resistance, steatosis and histological fibrosis grades [22]. Role for decreased skeletal muscle myokines involved in FFA metabolism [138]. Potential therapeutic target via the myostatin and/or activin/follistatin axis [139]. Can occur alone or combined with O-MAFLD, requiring double therapeutic approach
GAFLD	Genetics-associated fatty liver disease	Genetic variants either monogenic or polygenic not necessarily causing metabolic abnormalities [140]
LAFLD	Lipodystrophy-associated fatty liver disease	Fat or muscle mass abnormalities with partial or complete loss of adipose tissue and predisposition to develop fatty liver without obesity. Classified as congenital, familial partial or acquired disorders [84, 141]
AAFLD	Alcoholic-associated fatty liver disease	Occurs with significant chronic alcohol consumption. Similar potential progression as for NAFLD and risk of associated metabolic/cardiovascular complications [50, 84, 142]
CAFLD	Combined causes of FLD	Overlap of different entities of FLD, e.g., obesity and sarcopenia or genetic causes, or lipodystrophy
XAFLD	Yet-to-be-defined subgroups	Conditions of FLD where etiology remains (temporarily or constantly) undefined (e.g., autoimmune hepatitis [143]; viral hepatitis [144]), or plays an emerging role (e.g., thyroid subclinical dysfunction [145], mitochondrial dysfunction [40, 82, 146, 147]

As for MAFLD, however, any sub-classification linked to liver steatosis needs validation studies with terminology to be agreed upon [84]. While searching for better classification of liver steatosis, both researchers, clinicians, scientific societies, patients’ associations, and other stakeholders must be aware that change in terminology requires a better understanding of the molecular basis of the disease entity. Benefits of patients must be balanced along with novel risk stratification and characteristics of disease [87]. 

### Conclusions and future perspectives

The rapid epidemiological increment and the global diffusion of NAFLD is a matter of major concern in terms of healthcare and social burden of disease. Both etiology and pathogenesis of NAFLD are largely unknown, and a multidisciplinary approach is required to handle a frequent liver disease still missing a definitive therapy, beside lifestyle, and maintenance or achievement of ideal body weight. The ongoing discussion urges to revise the terminology [11, 12] since the acronym NAFLD has several limitations: it suggests ignorance about true etiology, it remains an exclusion criterium, it can be ambiguous and misleading, and appears to stigmatize the affected individuals because of the word “alcoholic”. Since NAFLD is very often and closely associated with metabolic dysfunctions, the current view is to shift the acronym NAFLD to MAFLD, i.e., metabolic-associated fatty liver disease. MAFLD becomes an “active” diagnosis based on the presence of overweight/obesity or, in the lean subject, on the combination of metabolic dysfunctions which act as high‐risk factors for events. MAFLD is independent of alcohol intake and the co‐existing causes of liver disease. The change in terminology is likely to improve the classification of affected individuals, the disease awareness, the comprehension of terminology and pathophysiological aspects involved, the choice of more personalized therapeutic approaches, while avoiding the intrinsic stigmatization due to the term “non-alcoholic” (Table 4).

**Table 4:** Principal differences between NAFLD and MAFLD

Major features	NAFLD	MAFLD
Initial diagnosis of liver steatosis	Imaging Histology	Imaging Histology Biomarkers
Terminology criteria	Based on “negative” assumptions	Based on “positive” assumptions
Terminology comprehension/disease awareness	Poor	Clear
Terminology stigma	Yes (“alcoholic”)	No
Alcohol intake criterium	Dependent	Not dependent
Metabolic dysfunction	Not required	Required
Combination with other liver diseases	No (excluded)	Possible, if present
Liver biopsy	Essential for diagnosis of NASH	Not required
Chance for multidisciplinary interactions	Medium	Maximum
Therapeutic reflections	Few	Several
Possibility to identify groups at increased risk	Moderate	Likely high for hepatic/extrahepatic complications (metabolic, cardiovascular, genetic risk) Limited data in pediatric populations

Even more recently, other sub-classification have been proposed, e.g., the LDE terminology [128], and a detailed nomenclature has been proposed to concentrate the heterogeneous causes of fatty liver disease under one umbrella [84] (Table 3). We must take note of such shifts of paradigm and contribute to advance the discussion further. Several partners must agree upon a novel terminology, including clinicians, researchers, pharmaceutical industries, patients and their associations, and scientific societies. In this multidisciplinary field, we need motivated and dedicated researchers with holistic views, to bring tangible pathophysiological, diagnostic, and therapeutic benefits for the populations worldwide suffering from fatty liver disease and related burden of disease. Along with this shift of paradigm, i.e., NAFLD vs. MAFLD, the role of internal medicine and internists is undoubtedly gaining even more trust.

## Supplementary Information

Below is the link to the electronic supplementary material.

Supplementary file1 (DOCX 33 KB)

## Data Availability

Data sharing not applicable to this article as no datasets were generated or analysed during the current study.
