# Correction to: NAFLD, MAFLD, and beyond: one or several acronyms for better comprehension and patient care

**DOI:** 10.1007/s11739-023-03252-5

**Published:** 2023-03-15

**Authors:** Piero Portincasa

**Affiliations:** Clinica Medica “A. Murri”, Department of Preventive and Regenerative Medicine and Ionian Area (DiMePrev-J), University of Bari Aldo Moro, Piazza Giulio Cesare 11, 70124 Bari, Italy

**Correction to: Internal and Emergency Medicine** 10.1007/s11739-023-03203-0

In the original article under the section NAFLD and metabolic dysfunctions the word "painful" was incorrectly written as "painless".

Also, the small gramatical error "are" changed to "is" under the section Conclusions and future perspectives.

The original article has been corrected.

