# Supplementary material for: NAFLD, MAFLD, and beyond: one or several acronyms for better comprehension and patient care
Source: Intern Emerg Med. 2023 Feb 17;18(4):993–1006. doi: 10.1007/s11739-023-03203-0 (PMC10326150; doi:10.1007/s11739-023-03203-0)
Supplement: Supplementary file 1 — Supplementary file1 (DOCX 33 KB) [file 11739_2023_3203_MOESM1_ESM.docx]

*IAEM-Review*

18.01.2023

**NAFLD, MAFLD, and beyond. One, or several acronyms for better comprehension and patient care.**

**Piero Portincasa**

Clinica Medica “A. Murri”, Department of Preventive and Regenerative Medicine and Ionian Area (DiMePrev-J), University of Bari Aldo Moro, Bari, Italy

**SUPPLEMENTARY MATERIAL**

**Supplementary Table 1. Causes of fatty liver disease (FLD)**

| - Non-alcoholic fatty liver disease (NAFLD) |
| --- |
| - Alcoholic liver disease (ALD) |
| - Hepatitis C (in particular genotype 3) |
| - Lipodystrophy |
| - Wilson disease |
| - Starvation |
| - Parenteral nutrition |
| - Abetalipoproteinemia |
| - Drugs (e.g., methotrexate, tamoxifen, glucocorticoids, amiodarone, valproate, anti-retroviral agents for HIV) |
| - Acute fatty liver of pregnancy |
| - HELLP (Haemolytic anaemia, Elevated Liver enzymes, Low Platelet count) syndrome |
| - Reye syndrome |
| - Inborn errors of metabolism (lecithin-cholesterol acyltransferase [LCAT] deficiency, cholesterol ester storage disease, Wolman disease) |
| - Drug-induced liver disease (DILI) |
| Adapted from Grattagliano I, Di Ciaula A, Baj J, Molina-Molina E, Shanmugam H, Garruti G, Wang DQ, and Portincasa P (2021). Protocols for Mitochondria as the Target of Pharmacological Therapy in the Context of Nonalcoholic Fatty Liver Disease (NAFLD). Methods in molecular biology 2310, 201-246; doi 10.1007/978-1-0716-1433-4_12. |
